# A Space-Division Multiplexing Method for Fading Noise Suppression in the Φ-OTDR System

**DOI:** 10.3390/s21051694

**Published:** 2021-03-01

**Authors:** Yixin Zhang, Jingxiao Liu, Fei Xiong, Xuping Zhang, Xiaohong Chen, Zhewen Ding, Yunyin Zheng, Feng Wang, Mengmeng Chen

**Affiliations:** 1 College of Engineering and Applied Sciences, Nanjing University, Gulou District, Nanjing 210093, China; zyixin@nju.edu.cn (Y.Z.); 18096617737@163.com (J.L.); xpzhang@nju.edu.cn (X.Z.); DG1834002@smail.nju.edu.cn (X.C.); dingzhewen@gmail.com (Z.D.); yuchi1114@sina.com (Y.Z.); wangfeng@nju.edu.cn (F.W.); 2 Key Laboratory of Intelligent Optical Sensing and Manipulation, Ministry of Education, Nanjing University, Nanjing 210093, China; 3 Inner Mongolia Electric Power Survey & Design Institute Co., Ltd., Hohhot 010011, China; xfei@nmdsy.com; 4 College of Electronic and Optical Engineering & College of Microelectronics, Nanjing University of Posts and Telecommunications, Nanjing 210046, China; 5 School of Electronic Engineering, Nanjing XiaoZhuang University, Nanjing 211171, China

**Keywords:** phase-sensitive time-domain reflectometry (Φ-OTDR), fading suppression, space-division multiplexing (SDM), multi-core optical fiber (MCF)

## Abstract

Phase-sensitive time-domain reflectometry (Φ-OTDR) can be used for fully distributed long-distance vibration monitoring. There is a fading phenolmenon in the Φ-OTDR, which will cause the signal intensity somewhere to be too low to extract the phase of the signal without distortion. In this paper, the Φ-OTDR based on space-division multiplexing (SDM) is proposed to suppress fading and we used multi-core optical fiber (MCF) to realize SDM. While inheriting the previous optimization strategy, we proposed a strategy based on frequency spectral similarity to process multiple independent signals obtained by SDM. And we compared the two methods. Through the experiments, the distortion rate can be reduced from an average level of 9.34% to less than 2% under continuous running of 270 s, which proves that SDM is a reliable technical route to achieve fading suppression. This method can effectively improve the fading suppression capability of the existed commercial systems.

## 1. Introduction

Phase-sensitive optical time-domain reflectometry (Φ-OTDR) has been widely applied in various applications such as structural health monitoring [1,2,3,4], intrusion detection [5,6] and geological hazard monitoring [7,8] due to its high sensitivity and distributed measurement ability. In recent years, it has also been innovatively proposed to be used in the field of optical networks [9,10]. Φ-OTDR uses an ultra-narrow linewidth laser as the system light source, and utilizes the interference effect between the Rayleigh backscattered (RBS) light generated by multiple scattering positions within the pulse width of the inject light in the optical fiber to increase its sensitivity to external disturbances. It has the ability to demodulate the phase of RBS to obtain the disturbance information carried by RBS. Once the fiber is disturbed, vibration around the optical path can cause phase modulation and the RBS phase variation has a linear response to the disturbance. Therefore, high-fidelity reconstruction of the disturbance signal along the fiber can be achieved through demodulating the RBS phase.

Nevertheless, the amplitude of RBS trace is jagged [11]. Interference among a large amount of backscattered light generated at different positions gives rise to a new kind of problem called fading noise [12]. Rayleigh fading noise (RFN) is fluctuations in backscattered signals, which causes some low intensity areas which may be close or even lower than system noise floor [13], and it makes the system difficult to demodulate the phase signal properly in such low intensity areas resulting in false monitoring [14]. Fading effect adds a key noise term limiting to the signal-to-noise ratio (SNR) of the Φ-OTDR system [15,16].

In recent years, many researchers have focused on methods for RFN reduction [17,18]. Using multiple independent detection channels could be helpful [12]. Thus, multi-frequency technology was proposed. In 1992, Shimizu et al. [13] studied the characteristics of fading noise in coherent optical time-domain reflectometry (C-OTDR) and derived the effects of frequency shift averaging (FSA) on fading noise reduction, but this method would not be suitable for dynamic measurement. Another way to suppress the fading was proposed by Tao et al. in 2018 [19]. They described an Φ-OTDR with a multi-frequency nonlinear frequency modulation (NLFM) optical pulse. They compressed the RBS generated by continuous wave (CW) light with non-linear tuning frequencies through signal process. This method can obtain higher probe pulse energy without sacrificing spatial resolution and will increase the overall SNR to reducing the possibility of fading. However, in long-distance measurement, the fading was still a problem due to the attenuation of the RBS intensity at the sensing fiber tail. Besides these methods, many scholars have put forward and verified the feasibility of frequency-division multiplexing (FDM). In 2001, Mermelstein et al. calculated and measured the frequency difference condition when two probe signals are statistically independent [20], and these results server as a guidline for the development of Φ-OTDR with FDM structure. Then in 2013, Pan et al. [21] brought forth a multi-frequency Φ-OTDR with equal frequency interval of several tens of MHz using phase modulator. This method introduces multiple frequency signals to avoid fading in space domain since the location of fading areas are different for independent frequencies. Based on this idea, Hartog et al. [18] described an Φ-OTDR that allows the measurement to be carried out quasi-simultaneously at multiple probe frequencies and used it for seismic wave detection. They reduced the noise caused by fading and improved the SNR by aggregating the data obtained. Due to the short time of interference caused by seismic wave, the problem of long-time signal acquisition was not considered in this article, and there was no problem of dynamic selection through multiple measurement results. No further discuss on the phase difference signal behavior in the fading region was performed. In 2019 Zabihi et al. [22] used three different probe frequencies in Φ-OTDR based on the FDM principle. They realized signal reconstruction by a tracking algorithm for selecting the optimum probe signal at any time continuously. However, in the commonly used FDM method, the structure of system is relatively complex due to the need to obtain multiple frequencies, and the bandwidth of detection signal becomes larger, which increases the difficulty of system hardware implementation.

In some applications, such as seismic wave monitoring, aerospace vehicle monitoring and other heavy equipment monitoring, much attention is paid to improve the sensing performance, as the accuracy of monitoring is important. It is an important issue to suppress fading noise. When using multi-frequency technology for fading suppression, it would be inevitably to change the system structure, which complicates the hardware design. Therefore, we hope that there is a way to improve the fading noise suppression performance based on the existed system structure. Due to the characteristics of random refractive index fluctuations, it is considered that multiple independent measurement samples could also be obtained through space-division multiplexing (SDM). If SDM could be combined with Φ-OTDR, it could provide a new way to suppress fading in Φ-OTDR.

In this article, we tried to effectively suppress the fading noise in Φ-OTDR without changing the structure of the existed sensing system. To achieve this purpose, we proposed an Φ-OTDR using multi-core fiber (MCF) to achieve SDM. On the one hand, although the price of MCF is a little higher than single-mode fiber (SMF), it is acceptable for relative precision applications. On the other hand, with the maturity of MCF production technology and the increase in market demand [23], its cost will gradually decrease [24,25]. Through the S-type series connection of fan-in and fan-out, we achieved the object of not changing the hardware structure of sensing systems. In addition, to predict the fading phenomenon and choose the optimum core which gives the best phase signal at any moment, we proposed a new optimization algorithm called Maximum spectrum similarity selection (MSSS) besides inheriting the original algorithm [22]. In this way, we could suppress the fading noise only by changing the sensing fiber and improving signal processing method. 

The rest of this paper is structured as follows: Section 2 introduces the principle of fading noise and our method. Section 3 describes the experimental setup. Section 4 presents the results of our experiments which prove the feasibility of this method, and a discussion. Finally, Section 5 draws the conclusions.

## 2. Principle

**Figure 1:**
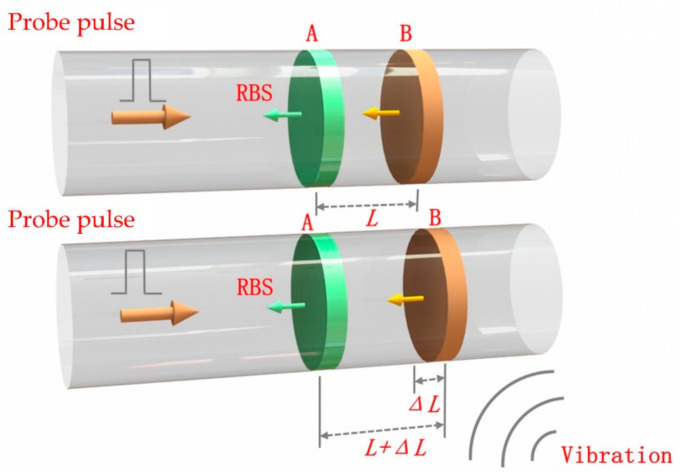
Principles of demodulating the phase information of rayleigh backscattered (RBS) light.

With the orthogonal demodulation and the phase unwrap, the phase can be extracted from the in-phase and the quadrature components of the beat signals [26]. Then we use differential methods to reconstruct vibration signals [27]. Figure 1 shows how an external vibration that induces extra stress on the fiber and results in a change in optical path length (OPL), where two segments of fiber A and B with a length of *L* are selected as the reference regions. The change in length *ΔL* is directly related to the change in relative phase difference *Δφ* between the two regions. Any external perturbation within two specific points changes the phase of the backscattered light wave. Therefore, the reconstruction of external vibration signal can be realized by demodulating the phase difference *Δφ*.

(1) $$\Delta \varphi =\frac{4\pi n}{\lambda }\Delta L$$

where *n* is the refractive index of fiber; *λ* is the wavelength of probe light; *ΔL* is fluctuation of fiber length results from external perturbation.

**Figure 2:**
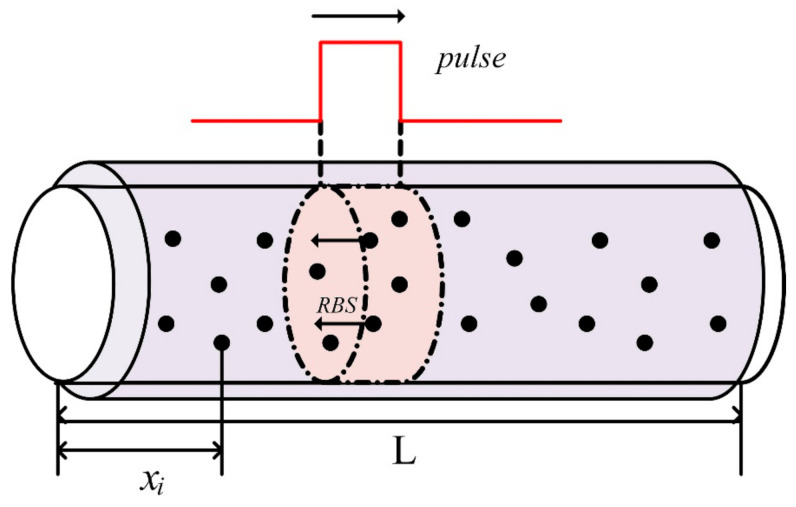
1-D scattering model Schematic diagram.

However, the above description is an ideal situation. In practice, due to the existence of fading, the amplitudes of regions A and B will be very low and even submerged in noise, which will affect the phase detection result. According to the one-dimensional (1-D) scattering model, tiny refractive index fluctuations in fibers can be seen as scattering points and the RBS waveform is closely related to the characteristics of scattering points [28]. Figure 2 shows 1-D scattering model, we assume that in a fiber segment of length L, the number of randomly distributed scattering points is *N*, and the scattering points are marked as 1, 2… *n* along the distribution distance. *x_i_* represents the distance of the *i_th_* scattering point in the fiber from the beginning of the fiber. Electric field of scattered light at time t is *E(t)*. This is the superposition of scattering signals in the range of optical pulse at a certain position of optical fiber and these points are numbered as [*x_a_*, *x_a+1_* … *x_b_*]. The fiber loss within pulse duration can be negligible owing to the pulse width is generally narrow [13], so *E(t)* can be simplified described as:

(2) $$E\left(t\right)=E_{0}e^{-\alpha v_{g}t}\overset{b}{\underset{i=a}{\sum }}\sqrt{\xi_{i}}\cos\left(2kx_{i}-\omega_{0}t+\varphi_{0}\right)$$

*E*_0_ is electric filed intensity of injected pulse. *α* is the loss coefficient of fiber. *v_g_* is the speed of light propagating in optical fiber. *ξ_i_* is the scattering coefficient of *i_th_* scattering point. *k* is wave number of light in fiber. *ω*_0_ is the angular frequency of the probe pulse. *φ*_0_ is the initial phase of injected pulse.

In the heterodyne detection structure, RBS returning from fiber is mixed with the optical local oscillator (OLO). The electric filed intensity of OLO can be expressed as:

(3) $$E_{lo}\left(t\right)=E_{l}\cos\left(\omega t+\varphi_{l}\right)$$

*E_l_* is electric filed intensity of injected continuous wave (CW). *ω* is the angular frequency of CW. *φ_l_* is the initial phase of CW. The photodetector detects the optical power, and its output photocurrent can be given as the following formula:

(4) $$\begin{array}{ll} I\left(t\right)\propto {\left(E\left(t\right)+E_{lo}\left(t\right)\right)}^{2}\\ & =\frac{1}{2}E_{0}^{2}e^{-2\alpha \nu_{g}t}\sum_{i=a}^{b}\xi_{i}+E_{0}^{2}e^{-2\alpha \nu_{g}t}\sum_{j>i}^{b}\sum_{i=a}^{b}\sqrt{\xi_{i}\xi_{j}}\cos\left[2k\left(x_{i}-x_{j}\right)\right]\\ & +E_{0}^{2}e^{-2\alpha \nu_{g}t}\sum_{j>i}^{b}\sum_{i=a}^{b}\sqrt{\xi_{i}\xi_{j}}\cos\left[2k\left(x_{i}+x_{j}\right)-2\omega_{0}t+2\varphi_{0}\right]+\frac{1}{2}E_{l}^{2}+\frac{1}{2}E_{l}^{2}\cos\left(2\omega t+2\varphi_{l}\right)\\ & +E_{l}E_{0}e^{-\alpha v_{g}t}\sum_{i=a}^{b}\sqrt{\xi_{i}}\cos\left(2kx_{i}-(\omega_{0}-\omega )t+\varphi_{0}+\varphi_{l}\right)\\ & +E_{l}E_{0}e^{-\alpha v_{g}t}\sum_{i=a}^{b}\sqrt{\xi_{i}}\cos\left(-2kx_{i}+(\omega_{0}+\omega )t+\varphi_{l}-\varphi_{0}\right) \end{array}$$

Due to the limitation of the measurement bandwidth of photodetector, the double optical frequency term and optical frequency superposition term can be ignored. The first and fourth term is direct current (DC) term, and the second and sixth term is alternating current (AC) term which represents the interference between backscattering light and the interference between RBS and OLO, respectively, are used to perceive external distribution events. According the formula, characteristics of scattering points influence the backscattering signal received by the photodetector. Due to the inhomogeneous spatial distribution of effective index, the amplitude of RBS conforms to a Rayleigh distribution [29], forming a jaded appearance in the backscatter measurement. 

Therefore there is a certain probability that RBS will fall into the weak zone. When RBS falls into the weak zone, SNR of the signal amplitude will decrease. Intensity noise will transfer to phase noise during phase demodulation [30]. When we use the method of signal difference between two nearby reference regions A and B to demodulate the phase, the value of phase SNR is associated with backscatter power of two position which is selected to make differencing, is given by:

(5) $$SNR_{\phi }=\frac{\sigma_{\phi }^{2}}{\sigma_{n}^{2}\left[1/A^{2}\left(t_{1}\right)+1/A^{2}\left(t_{2}\right)\right]}$$

where $\sigma_{n}^{2}$ and $\sigma_{\phi }^{2}$ are the variance of intensity noise and external disturbance separately; *A(t1)* and *A(t2)* represent to the signal amplitude at two different locations where are selected for a differential process [30]. It can be seen the SNR of phase will raise with the increase of RBS amplitude, so fading will have an adverse effect on phase demodulation.

Since fading is closely related to the characteristics of scatters, obtaining multiple independent measurement samples can be an effective fading suppression method. In the process of optical fiber manufacture, doping, drawing and other process will inevitably cause random fluctuation in the core refractive index distribution along the lateral direction of the fiber. The magnitude of these refractive index fluctuations is much smaller than the wavelength of the incident light, so the positions of such refractive index fluctuations can be approximated as discrete scattering points in space [12]. In optical fiber, scatters are randomly distributed over distance, and the scattering rate of each scattering points is also random. Accordingly, the intensity fluctuation which is the result of interference of scattered light generated by millions of scattering points would also be random. It can be inferred that, for different batches of optical fibers, even different sections of the same batch of optical fibers, the fluctuation of fading has very good independence. Therefore, one of the ways to obtain independent measurement can be to use the signal from different fiber channels.

**Figure 3:**
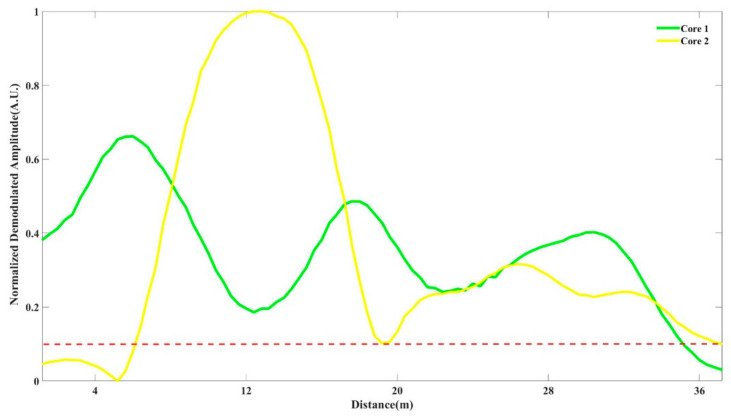
Signal amplitude fluctuation in different fiber channels.

If the optical pulses are injected into multiple fiber channels, the phase term in the formula would change due to the inconsistent characteristics of the scattering points of the fiber, *x_i_* and *ξ_i_* are not exactly the same, so we estimate that the curve amplitude fluctuation of Φ-OTDR only depends on the fiber state in the case of the optical frequency and the shape pulse having been determined. When we obtain the backscattering signals from multiple fiber channels, the fluctuation of RBS is different for each channel. As Figure 3 shows, the yellow solid line and the green solid line are the normalized demodulated amplitude of RBS in two dependent cores. The horizontal red dotted line shows a threshold which we assumed is located at 0.1 of the normalized result. To achieve a good SNR of demodulation phase, the threshold needs to guarantee an empirical value of at least 10 dB SNR at the output for areas above it [22]. For the commercial system used here, a SNR more than 10 dB could be obtained when the signal strength exceeds 10% of the entire quantization range through preliminary calibration [31]. Obviously, the locations of fading in two RBS amplitude curves are not exactly the same. It can be inferred that probability is low when the fading areas in the signal appear simultaneously at the same location for the RBS curve obtained with different cores.

After solving the source of independent measurement, the next key problem is how to continuously optimize multiple measurement results. According to the characteristics of fading, we proposed two different optimization algorithms for dynamic selection among multiple sets of signals to achieve the reconstruction of vibration signals. One is to dynamically select the demodulation results corresponding to the signal with the strongest amplitude among multiple backscattering signals, inherited from the work mentioned in our previous works [22]. The other is to dynamically select the best two groups of the results based on frequency domain characteristics and average them to acquire the reconstructed vibration signals. Here, we used spectral similarity for data selection. If the quality of reconstructed signal is good, its frequency spectrum will have a high degree of similarity. This is because noise has random characteristics, if reconstructed signal contains a lot of noise, its spectrum similarity would be low. When using frequency spectrum for similarity comparison, we can make flexible bandwidth selection according to the characteristics of signal and noise. Here, the correlation coefficient is used to evaluate the similarity of frequency spectrum, calculated as follows:

(6) $$\rho \left(A,B\right)=\frac{1}{N-1}\overset{N}{\underset{i=1}{\sum }}\left(\frac{\bar{A_{i}-\mu_{A}}}{\sigma_{A}}\right)\left(\frac{B_{i}-\mu_{B}}{\sigma_{B}}\right)$$

Here, *μ_A_* and *σ_A_* are the mean and standard deviation of A, *μ_B_* and *σ_B_* are the mean and standard deviation of B. When we obtained the two sets of data with the highest spectral similarity at any time, we averaged them to obtain the final reconstructed signal. 

Obviously, these channels need to be consistent in their perception of the same external disturbance when using SDM in order to reconstruct the disturbance signal over time. Multi-fiber cables have variety of structures. In some structures, due to the outer armor and the loose structure of the inner fibers, the coupling of the external disturbance of each fiber may be inconsistent. When using multi-fiber cables, it is necessary to select the appropriate structure. Considering the structure between each fiber core for MCF is more compact than that of multi-fiber cable, so that the coupling consistency to external disturbances is higher, can achieve signal restoration and fidelity, so here we used MCF to perform experiments to verify the feasibility of SDM.

## 3. Experimental Setup

**Figure 4:**
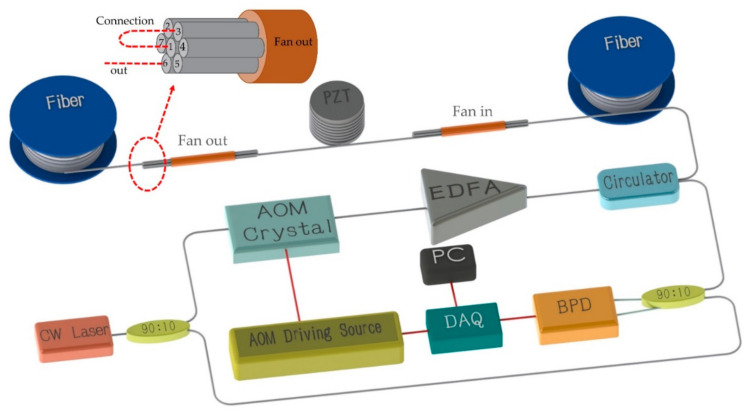
MCF assisted phase-sensitive time-domain reflectometry (Φ-OTDR) System setup. CW Laser: Continuous Wave Laser; AOM: Acousto-Optical Modulator; EDFA: Erbium Doped Fiber Amplifier; Cir: Circulator; SMF: Single-mode fiber; MCF: Multi-core optical fiber; PZT: Pizeo-electric Transducer; BPD: Balanced Photo Detector; DAQ: Data Acquisition.

**Figure 5:**
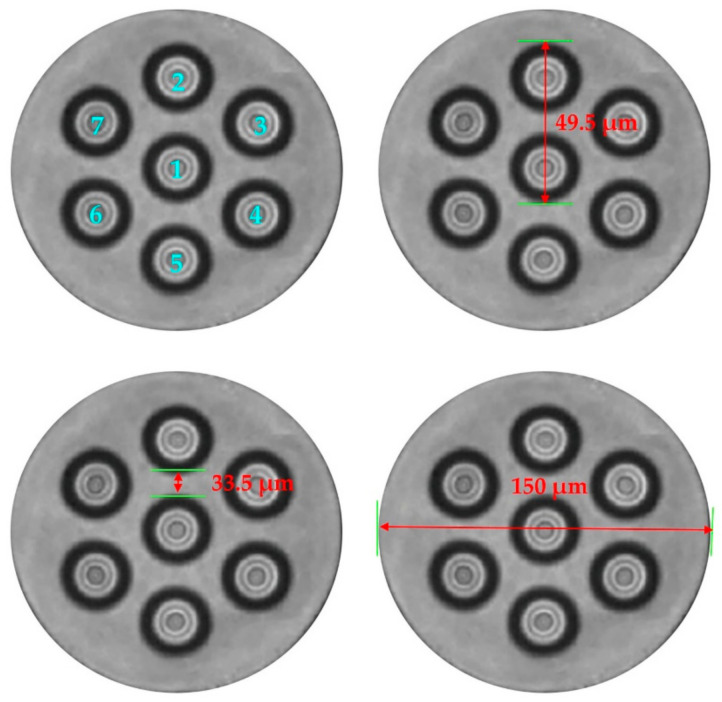
Cross section of MCF.

The experimental setup is shown in Figure 4. Light from a narrowband continuous wave laser with linewidth of 3.7 kHz operating at 1550 nm was divided into an interrogation arm and a local oscillator arm. The interrogation arm passed through an acousto-optical modulator (AOM) which created a probe pulse of 100 ns width, 1 kHz repetition rate and 200 MHz frequency shift. After the pulse was amplified by an erbium-doped fiber amplifier (EDFA), it was injected into the 200 m SMF through a circulator. The peak power of the EDFA output was about 18 dBm. Fan-in, fan-out was used to connect each core of MCF (YOFC, MC1010-A). The typical value of cross-talk in MCF fiber is −50 dBm/100 km, so inter-core crosstalk could be ignored in the measurements. MCF was wound on the piezoelectric transducer (PZT) in parallel in the experiment. The profile structure of the MCF used in experiment is shown in Figure 5. We can see that the core-core pitch is 41.5 ± 1.5 μm and the cladding diameter is 150 μm, therefore the arrangement of the cores is very compact. This ensures the consistency of the coupling. The six outer cores are distributed in a regular hexagon. Since this is a demonstrative experiment, we focused on the ability of using MCF to suppress the fading, rather than measuring distance and spatial resolution. We only used a 50 m MCF in the experiment. Due to the short length of MCF, we connected a section of SMF (200 m) to the front end of the MCF for extension, in order to ensure the integrity of the signal. Vibration events were applied to MCF. And disturbed area of MCF was about 25 m. The gauge length employed for phase demodulation was about 34 m in the subsequent data processing.

We numbered the cores in a MCF from one to seven to facilitate subsequent distinction. In order to achieve the measurement of multiple fiber core data without changing the existing sensing structure, we connected the cores in series end-to-end, so we could use only one photodetector to receive the signal based on this structure. The RBS returning from the fiber under test (FUT) was mixed at the receiver with local oscillator. A data acquisition (DAQ) system was used to capture the output signal with 250 MHz sampling rate. As a proof of principle experiment, we only chose three of the seven cores and connected them end-to-end. As shown in Figure 4, three fiber cores were connected end to end in an S-shape. We used PZT to simulate sinusoidal vibration signals of different frequencies. 

**Figure 6:**
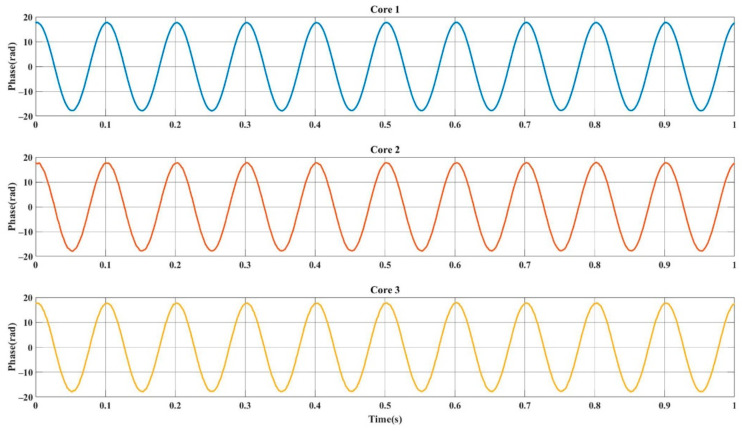
The result of three independent fiber cores.

A 10 Hz sinusoidal signal was applied on PZT. Figure 6 presents the demodulation results of three cores separately when there is no fading. These are the results that we selected from a large amount of data after demodulating the signals of the three cores in traditional way. It can be seen from the figure that under the same disturbance, the demodulation phase results of each fiber were good and had a high degree of consistency, we can expect these results due to the MCF as SDM. Therefore, for multiple sets of data, we could achieve an optimal trace by directly hopping from a distorted signal to a well-shaped one in the proper time. Here, we adopted two different algorithms for predicting the occurrence of distorted phase shape and compared the results of the two methods. Maximum amplitude selection (MAS) was based on signal amplitudes. Comparing the intensity of each group of signals at each moment and selecting the reconstruction signal corresponding to the highest intensity data as the reconstruction signal at that moment. The other way was based on frequency domain comparison, we called it as MSSS. We calculated the short-time Fourier transform (STFT) of each fiber demodulation result. In the confirmatory experiment, we only selected the two sets of data with the highest spectral similarity and averaged them to obtain the reconstructed disturbance signal.

**Figure 7:**
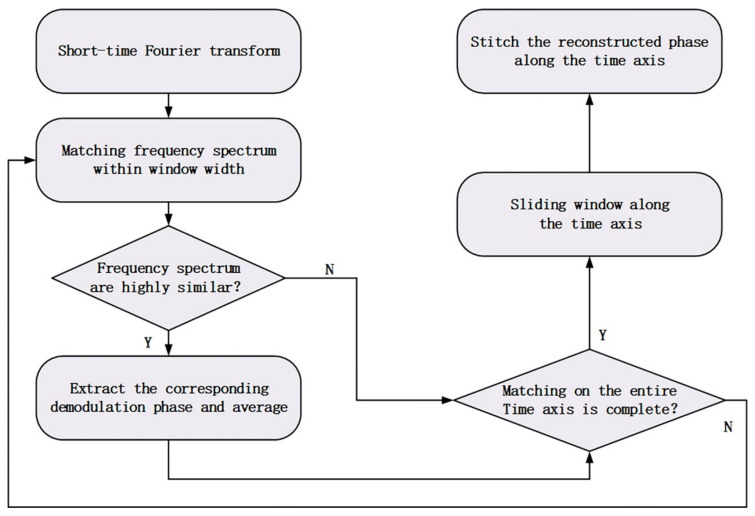
The process of judging using short-time Fourier transform (STFT) as a standard program.

The specific steps of MSSS algorithm is shown in Figure 7. We applied a short-time Fourier transform (STFT) on each group of data, compared the frequency spectrum in each time period which refers to the window width during the STFT, and obtained two groups of data with the highest similarity. The demodulation phases corresponding to the data with the highest similarity in this time period was averaged to obtain the reconstructed phase in this time period. By sliding the window along the time axis, the STFT frequency spectrum matching was achieved along the time dimension. Splicing the obtained phases, and finally realizing the phase reconstruction.

## 4. Results and Discussion

**Figure 8:**
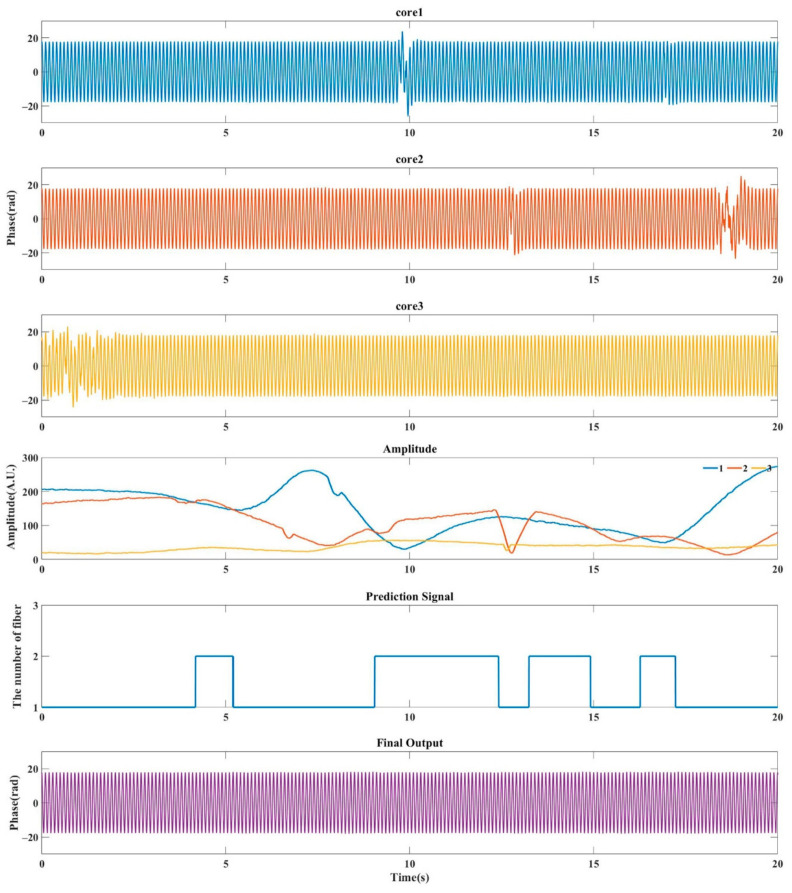
Extracted phase signal from all single beat frequency, as well as prediction signal and final output (selection based on signal amplitude).

**Figure 9:**
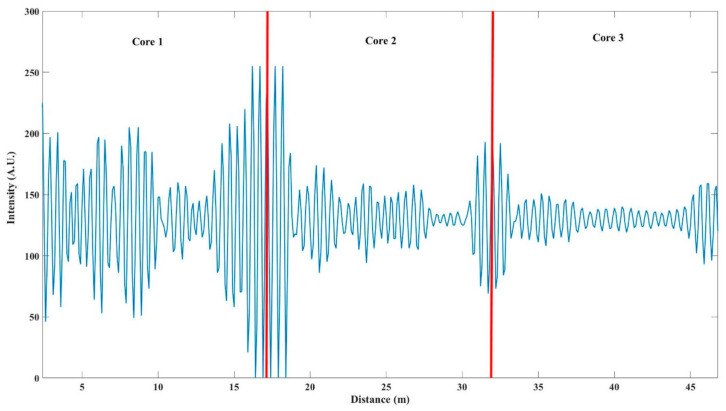
Intermediate frequency (IF) signal intensity of three cores.

Figure 8 illustrates the output for about 20 s with MAS algorithm. Due to the fact the amplitudes had some small fluctuations, we used averaging to mitigate the effect of these fluctuations. Here, we set the time window width for averaging to be 100 points, which corresponding to 0.1 s. Phase signals from core 1, core 2, core 3 and the final output show the reconstructed vibration signal obtained by demodulating each core separately, and the reconstructed signal obtained by the optimization algorithm. It can be clearly seen that extracted phases from all three cores have distortions for some different time ranges. The fourth line in Figure 8 is the lowest value of the signal intensity in the process of demodulating the three sets of RBS. Blue line is the demodulation amplitude of RBS in core 1, the red and the yellow line are the demodulated amplitude of RBS in core 2 and 3, respectively. Comparing the corresponding signal intensity, the distortion positions are all positions with weak amplitude. The fifth row in Figure 8 indicates the selection of data in the optimal algorithm. When the core corresponding to the highest amplitude in the RBS amplitude diagram changes, the system will jump to select the phase extracted from the core with the highest amplitude. As can be seen in this figure, since the intensity of core 3 was weak, the reconstruction results are basically selected from core 1 and 2. Due to the loss caused by fusion splicing process of MCF and fan-in/fan-out, the intensity of probe pulse would be attenuated during transmission and the loss of core 3 was larger than that of core 1 and core 2. Figure 9 shows the intermediate frequency (IF) signals in the three cores. It could be seen that with the increase of cascading degree, the intensity of IF signal decreases due to the connection loss. Core 3 has obvious attenuation. When we used signal strength as the selection criterion, we could avoid selecting the position with the weakest signal, thereby avoiding the distortion position. The final output is shown in Figure 8.

It can be seen in Figure 9 that the three cores were connected in series because the signal was continuous, while, it was worth noting that since the cores were cascaded in an S-shape, the signal of core 2 needed to be inverted during data processing to ensure that the corresponding positions of signals of three cores were consistent along the distance axis.

**Figure 10:**
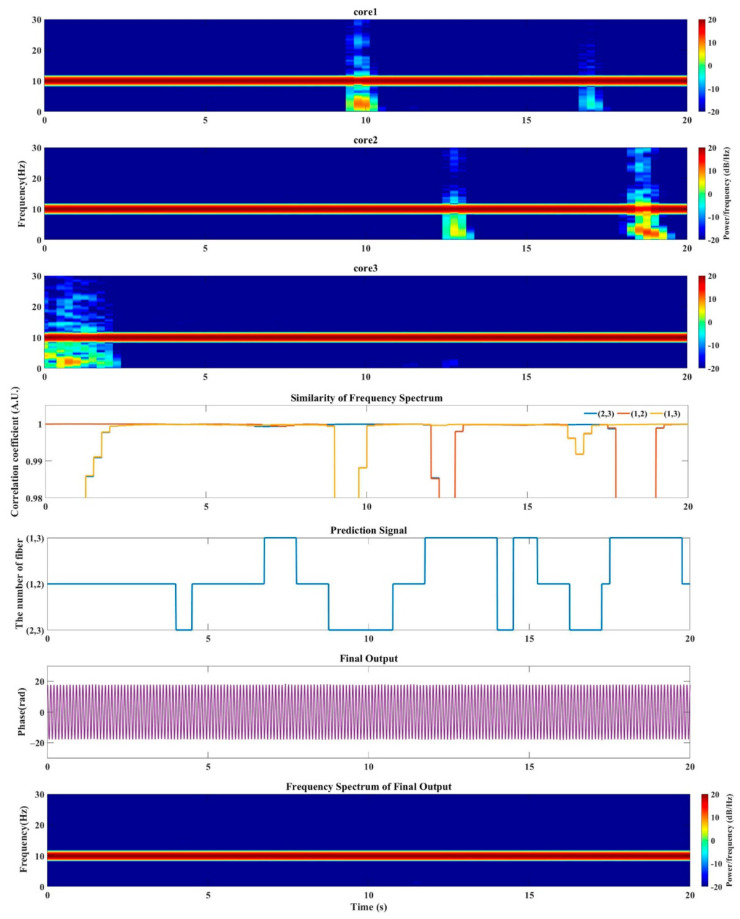
Extracted phase signal from all single beat frequency, as well as prediction signal and final output (selection based on frequency domain).

We also reconstruct the vibration signal with MSSS method to make a comparison. Figure 10 presents the output for 20 s. In order to show better frequency resolution, the window width of the STFT is 1 s, and the overlap of the sliding window is three-fourths of the window width. The subgraphs core 1, core 2 and core 3 in Figure 10 are the spectrograms corresponding to the extracted phases. Among them, the main energy is concentrated around 10 Hz, but at certain moments, the results obtained by the three cores have strong other frequency components. The fourth row in the Figure 10 shows the similarity of the pairwise comparison of the spectrum. The blue one is the spectrum similarity of the results obtained by cores 2 and 3, the red and yellow are the similarities of cores 1, 2 and cores 1, 3 respectively. By obtaining the maximum similarity at a unique moment, we can get the prediction signal, which is the curve shown in the next line. The prediction signal indicates the source of the signal extracted in the optimal algorithm at each moment, that is, the data obtained from which two cores are selected. The last two lines of graphs are the time domain and frequency domain graphs of the reconstructed signal obtained by the optimization algorithm. Obviously, this method also achieved an optimal trace, the range of distortion in extracted phases from all core 1, 2 and 3 was well avoided in the reconstruction results in Figure 10.

**Figure 11:**
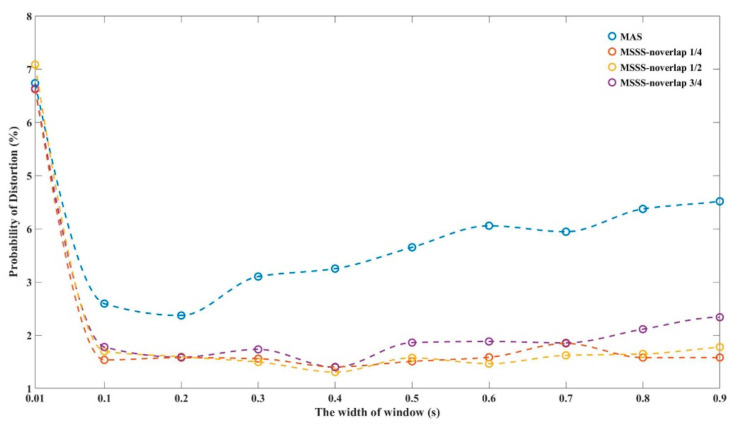
The probability of failure for different window width.

To further evaluate the impact of amplitude average window width in MAS and STFT window width in MSSS, we presented the probability of failure of the final output under the data length of 200 s for different window width (0.01 s~0.9 s) in Figure 11. Here, we assumed that any deviation of the peak-to-peak value of the demodulation phase from its peak-to-peak average that exceeds 10% is a failure. The probability of failure in the entire data is the distortion rate. In the STFT calculation, the overlap degree of the sliding window was involved. We also applied statistics on this and plotted it in Figure 11. The blue dotted line is the distortion rate fitting curve of MAS. The red, yellow and purple dotted line are respectively the fitting curve of the distortion probability of MSSS when the overlap is one-quarter, one-half and three-quarter of the window width. As the window width increases, the distortion rate decreases rapidly and then gradually increases in volatility. The distortion rate of MAS increases with window width faster than MSSS. For the curves under the three overlapping degrees, the inhibition effect is weak when the overlap is three-quarters, but the difference between the three is not very obvious. Overall, the overlap impact of MSSS seems to be small. The best point of the suppression effect in this figure is about 0.1 s. At this location, we obtained that the distortion rate of the extract phase of core 1, 2 and 3 is 7.08%, 7.72% and 15.96%, respectively. The output result after using MAS has a distortion rate of 2.59%, and the distortion rate of MSSS is 1.53%. In STFT, the size of window determines the time resolution and frequency resolution of the spectrum. The longer the window width, the higher the frequency resolution and the lower the time resolution after Fourier transform. When the window width is too small, it is easy to be affected by random noise. When the window width is too large, it would be unable to make timely response to the fading change. Therefore, the window width needs to be adjusted reasonably according to the requirements of frequency and time resolution. For the two methods, especially under the large window width, the suppression effect of the MSSS is obviously better than that of MAS.

**Figure 12:**
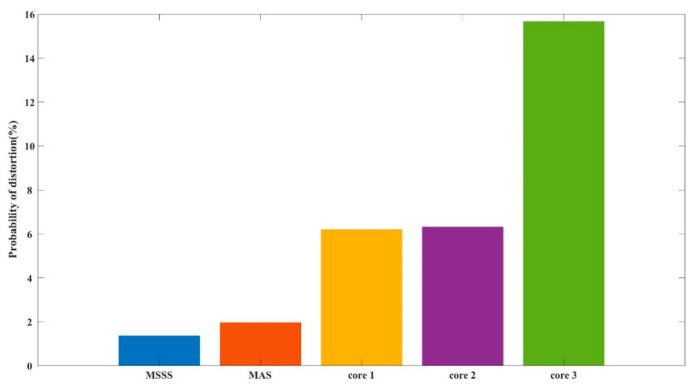
The distortion probability of demodulation results.

We collected data for about 270 s continuously. The vibration signal applied to PZT is a 10 Hz sinusoidal wave. According to the above results, we selected a window width of 0.1 s to perform statistics on the entire segment of data. The distortion rate of the extract phase of fiber 1, 2 and 3 is 6.20%, 6.32% and 15.66%, respectively. The output result after using MAS has a distortion rate of 1.97%, and the distortion rate of MSSS is 1.36%, as shown in Figure 12.

**Figure 13:**
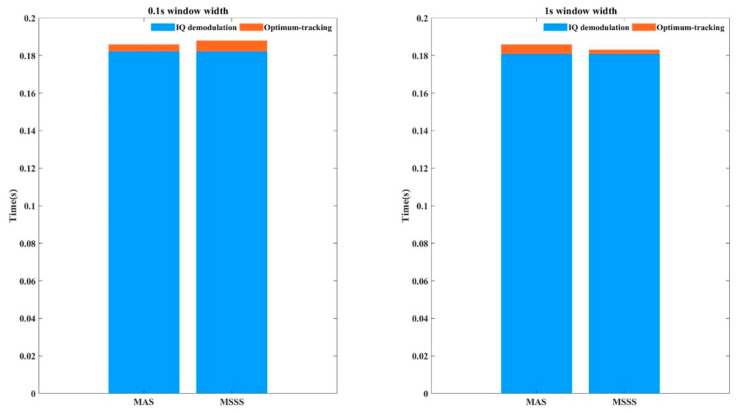
Algorithm time statistics.

Although the suppression effect of MSSS is better than MAS in most cases, its computational complexity is significantly higher than that of MAS. In order to further evaluate the influence of these two algorithms on the calculation pressure of global signal processing, we had counted the computing time of each part of the program in an environment where Central Processing Unit (CPU) is an Intel (R) Core (TM) i5-3470 CPU @ 3.20 GHz and Random-Access Memory (RAM) is 8.00 GB. (Dell, Xiamen, China). To verify the feasibility of the algorithm, the CPU was used for calculation. Our program was written in MATLAB and precompiled. The running time of each part is shown in Figure 13. When processing 200 s of data of the same length, in-phase/quadrature (IQ) demodulation takes about 36 s. When the window width is 0.1 s, the preferred tracking algorithm MAS takes about 0.7 s, and algorithm MSSS takes about 1.1 s; when the window width increases to 1 s, MAS takes about 0.9 s, and MSSS takes about 0.4 s. For the convenience of display in the figure, we gave the respective time consumption per unit time data. It can be seen that as the window width increases, the advantage of calculation amount of MAS will be weakened, even worse than that of MSSS. However, the increase of the window width will weaken the fading suppression effect. Therefore, in actual application, the two methods have their own advantages and disadvantages, but they both can achieve effective fading suppression, and the structure is simple.

This work demonstrated Φ-OTDR system with a simplified design of using MCF for SDM to suppress fading noise. For multiple sets of data obtained through MCF, optimization is required. In the optimization algorithm, in addition to the amplitude tracking in the previous work, this paper also proposed a spectrum similarity based optimum-tracking, which had achieved excellent fading noise suppression effects. When the window width is small, the calculation amount of MSSS is significantly higher than that of MAS. The calculation time of MSSS is about 1.5 times that of MAS when the window width is 0.1 s. The selection of algorithms in practical applications involves the trade-off between fading noise suppression effects and hardware capabilities. 

When the measurement length is further extended, the calculation pressure of the CPU will inevitably increase. And its calculation amount will increase exponentially as the number of cores increases. At this time, the choice of Graphics Processing Unit (GPU) will be an excellent technical route. For the promotion in practical applications, due to the high expense, there are still limitations in application temporarily using MCF. If a multi-fiber cable is used, in order to ensure the uniformity of the coupling of optical fiber to external disturbance, the optical cable structure needs to be considered. It should be able to calculate the coupling coefficient and correct the phase to the same, to facilitate subsequent data processing. The proposed method is quite likely to be used in engineering practice in the near future.

## 5. Conclusions

The Φ-OTDR system is improved based on the SDM structure and the optimum-tracking algorithm. In order to ensure the uniformity of the coupling of optical fiber to external disturbance, SDM is realized by MCF, and the distortion rate can be reduced from an average of about 9.4% to less than 2% under continuous time running for the proposed scheme. Compared with FDM, the proposed method does not need to change the hardware structure of the instrument of the sensing system, which offers a reliable option to achieve fading suppression for Φ-OTDR system. This method can be applied to the existed commercial systems to improve the fading suppression ability effectively. As the cost of MCF decreases, SDM will become a very practical technology to improve the SNR of Φ-OTDR system.

## Data Availability

All original data, vibration data and code will be made available on request to the correspondent author’s email with appropriate justification.
